# Prevalence of stress, anxiety and depression in with Alzheimer caregivers

**DOI:** 10.1186/1477-7525-6-93

**Published:** 2008-11-06

**Authors:** Maria Ferrara, Elisa Langiano, Tommasina Di Brango, Elisabetta De Vito, Luigi Di Cioccio, Claudia Bauco

**Affiliations:** 1 Department of Motor Science and Health, University of Cassino, Italy; 2 UVA (Unità Valutativa Alzheimer) "Dottore Angelico" di Aquino, Italy

## Abstract

**Background:**

Alzheimer's disease presents a social and public health problem affecting millions of Italians. Those affected receive home care from caregivers, subjected to risk of stress.

The present investigation focuses on stress, anxiety and depression in caregivers.

**Methods:**

Data on 200 caregivers and their patients were collected using a specific form to assess cognitive, behavioural, functional patient (MMSE, and ADL-IAD) and caregiver stress (CBI). The relationship between stress, depression and disease has been assessed by means of a linear regression, logistic analysis which reveals the relationship between anxiety, stress and depression and cognitive problems, age, the patient's income.

**Results:**

The caregivers are usually female (64%), mean age of 56.1 years, daughters (70.5%), pensioners and housewives (30%), who care for the sick at home (79%). Of these, 53% had little time for themselves, 55% observed worsening of health, 56% are tired, 51% are not getting enough sleep. Overall, 55% have problems with the patient's family and/or their own family, 57% at work. Furthermore, 29% feel they are failing to cope with the situation as they wish to move away from home. The increase in the degree of anxiety and depression is directly proportional to the severity of the illness, affecting the patient (r = 0.3 stress and depression r = 0.4 related to CBI score). The memory disorders (OR = 8.4), engine problems (OR = 2.6), perception disorders (OR = 1.9) sick of the patient with Alzheimer's disease are predictive of caregiver stress, depression is associated with the presence of other disorders, mainly behavioural (OR = 5.2), low income (OR = 3.4), patients < 65 years of age (OR = 2.9).

**Conclusion:**

The quality of life of caregivers is correlated with the severity of behavioural disorders and duration of the Alzheimer's disease. The severity of the disease plays an important role in reorganization of the family environment in families caring for patients not institutionalised. It is important to promote measures to soften the impact that the patient has on the caregiver, and that, at the same time, improves the quality of life of the patient.

## Background

Dementia disorders are one of the most compelling problems of social and public health. Dementia usually leads to a marked decrease in the cognitive, mental and also physical skills of the affected person, who, over time requires an increased amount of care, aid and support. It affects approximately one million Italians and this number could double by 2020 [1,2], on account of the improvements in general health and increased average life expectancy. In Italy, the annual budget needed to support dementia patients amounts to almost € 50,000,00 and 60% is provided by family networks; the government aid programme for these patients, is approximately 20 billion Euros. Almost 70% of cases of cognitive deterioration, observed during aging are of the Alzheimer type [1]. Alzheimer's disease (AD) is a progressive degenerative disease, that involves the central nervous system (CNS), negatively affecting the memory, mind and behavioural processes, with consequent loss of the ability to perform normal everyday activities and, therefore, loss of self-sufficiency of the subject. Intense research is currently aimed at defining the cause of the disease: the most recent findings stress the multi-factorial aetiology of the disease. Age represents the most important risk factor; AD affects approximately 10% of subjects > 65 years of age, the majority being female (78%) [2]. The incidence of AD increases with increase in age; the prevalence being ~1% in the subjects between 60 and 64 years old, and doubles every 5 years after the age of 65, until reaching 40% in people > 85 years of age [1-3]. With progressive aging of the Italian population (as in other Western countries), the AD problem becomes even more dramatic [4].

The problem is not limited only to the patient's loss of independence, but also has repercussions on the relatives. In fact, the progressive cognitive deterioration as well as patient's loss of physical capabilities, increase the need of constant care. Almost all the affected patients resolve their own needs and receive their daily care at home, placing themselves in the hands of the caregiver. The Anglo-Saxon literature identifies such term as "one who gives care". The caregiver is, therefore, the person who takes daily care of the patient; the time dedicated to this activity is comparable to a standard working day and the hours employed, in this job, increase as the disease worsens. In the worst scenario, taking care of the patient becomes a full-time occupation for the caregiver. The consequences of this role are not negligible, and compared to subjects of the same age and to the average population, caregivers are more at risk of compromising both their physical health and subjective psycho-social well-being [5]. In Italy, the caregivers are usually relatives of the patient, defined as "informal" in order to distinguish them from the institutional caregivers. Informal caregivers who choose to have the patients living with them in their home (65%) are mainly females (78% are wives or daughters), aged between 45 and 60 years old and the majority are retired (31.95%) and, in most cases, housewives (27.9%) [1-6]. To take care of a patient with dementia is an extremely onerous task. Enduring the patient's changes in the levels of ability and behaviour can be very stressful, thus exposing the caregiver to the risk of depression and physical vulnerability, especially if they do not receive support from other family members, friends or society. The main cause of stress, for the caregiver, stems from the difficulties resulting from the patient's disturbing behaviour [7]. The deterioration of both cognitive and physical abilities, often associated with no cognitive symptoms, such as psychotic symptoms, depression and changes in behaviour, typical of this disease, result in a heavy burden on the caregiver, representing a severe health, economic and social problem.

It is, therefore, imperative to prevent this disease, or to limit the damage related to such a heavy toll on the caregiver, when the onset has already occurred.

Attention should be focused, in particular, on the detrimental effects that the caring for AD subjects has on the caregiver. To this end, a survey on AD patients' caregivers, enrolled in the Cronos Plan at the AD Evaluation Unit **"Dottore Angelico" **of Aquino (Lazio Region), was implemented, the aim of this study being to evaluate their stress, anxiety and depression status.

## Methods

A retrospective survey was carried out on 200 caregivers recruited by random selection. Data were collected by means of a carefully designed questionnaire which included specific instruments for cognitive and behavioural appraisal and evaluation of stress both in the patient and the caregiver. In particular, to define the cognitive state of the patients Mini Mental State Examination (MMSE), defined by Folstein was used [8]; for the behaviour and the functional activity the Activities of Daily Living (ADL) by Katz et al. [9] and the Instrumental Activities of Daily Living (IADL) by Lawton & Brody were also adopted [10]. To evaluate stress in the caregiver, the Caregiver Burden Inventory (CBI) by Novak and Guest [11] was used. The CBI is used not only in the evaluation of the burden of care, but also to analyse the multidimensional aspects. The questionnaire consists of 24 questions, divided in 5 sections; it is used to estimate different factors of stress: amount of time dedicated; psychological burden (feeling of loss of opportunity); physical burden (negative feelings about ones own health); social burden (feelings of bad relationships within the family and in the workplace) and emotional burden (negative feelings towards the patient) [11]. It is easy to complete and simple to understand. The interviews, in the present study, were carried out at U.V.A.

The Service was commenced in the year 2000 and, up to now, 768 records have been collected, of which 563 referring to patients effectively registered in the Cronos Plan; the remaining 205 are under assessment or Drop-Outs (no longer in the Plan) as they are seriously ill or refuse care.

### Patient consent and ethical approval

Caregivers who contributed to the research have been informed on the project and gave consent to processing of personal data (L. 675/96) and the implementation of diagnostic and therapeutic procedures related to pharmacological monitoring protocol "CRONOS "DM 20.07.2000".

### Analysis

A database, in Access format, was created and a statistical analysis was made with the statistics programme Epi Info, version 3.3. For the analysis of differences, the "chi-square" test was employed and a value p < 0.05 was considered statistically significant. The relationship between dependent variables (stress and/or depression) and independent variables (severity of disease) has been evaluated using a model of simple linear regression while logistic regression was employed to study the relationships between one outcome variable (degree of anxiety, stress and depression) with more specific variables (such as cognitive problems, physical disability, patients' age, financial situation etc).

## Results

The characteristics of patients and caregivers, in the study population, are outlined in Table 1. The results demonstrate that the change in the caregiver's lifestyle coincided with the time of the AD patient's need of care. In fact, 39% of the AD patients have to be constantly supervised and 42% need to be helped also when washing and dressing, and for eating, etc., being completely dependent upon the informal caregiver. Inadequacy of the caregiver develops in these situations: in fact, 53% of the caregivers declare that they have little time for themselves; they feel that their own social life has been influenced, in some way, whilst 59% feel emotionally drained. Furthermore, 55% of the subjects interviewed consider that their own health has been affected; 67% claim to be ill; 56% feel physically tired; while 51% do not get sufficient sleep. The survey showed that these situations create severe misunderstandings also within the family, which is demonstrated by the fact that 55% of the caregivers argue with the other members of the family and feel they are being criticized. 54% feel some resentment towards relatives who could help, but choose not to. At home, 53% of the sample feel that their relationship with their spouse changed, as well as that with their children (49%) and the siblings (42%). Moreover, the study revealed that 57% of the caregivers do not work as efficiently as they usually did. The entire situation has led to some resentment of the caregivers toward the patient. Nevertheless, very high percentages (between 70 and 95%) of caregivers claim that they are not ashamed of the patients and don't feel embarrassed by the behaviour of AD patients and do not feel uncomfortable when friends visit their home. Of those interviewed, 29% claim to be unable to accept the situation and wish to abandon their home. Mean values of MMSE, ADL and IADL of the patients and CBI of the caregivers are outlined in Table 2. At the same time as the survey on the caregivers, an assessment was made of the severity of the patients' pathological conditions: linear regression demonstrated that an increase in the degree of anxiety and depression is directly proportional to the severity of the disease in the patient under care. In fact, a direct association emerged between stress and the CBI score (r = 0.3, p = 0.002) and between depression and the CBI score (r = 0.4, p = 0.0004). The logistic regression model, instead, demonstrated that the consequences of the failure of superior cortical functions, such as memory loss (OR = 8.4; p = 0.003), movement restrictions (OR = 2.6; p = 0.009) and loss of perception (OR = 1.9; p = 0.05), are predictive of stress in the caregiver, whereas depression is closely related to severity of AD due to additional symptoms, prevalently behavioural problems (OR = 5.2; p = 0.0003), low income (OR = 3.4; p = 0.02), patient age < 65 years (OR = 2.9; p = 0.02).

**Table 1:** Study population.

**Patients**			**Caregiver**		
**Variables**		**Value**	**Variables**		**Value**

Sex (%)	M	22	Sex (%)	M	36
					
	F	78		F	64

Age	M	76.9 ± 5.3	Age (media ± ds)	M	59.4 ± 14.3
(media ± ds)					
		77.8 ± 7			56.1 ± 13.3
					
	F			F	

Education (%)			Education (%)		
Secondary		80	Secondary	School	48
Primary			Certificate		

Profession (%)			Profession (%)		
Housewife		70	Housewife		30
Farmer		80	Employee		30

Married (%)		66	Relationship Status (%)		
			• Spouse		27.4
			• Children		70.5

-----------------			Living with patient (%)		79

-----------------			Sufficient Economical Conditions (%)		75.8

**Table 2:** Median values of MMSE, ADL and IADL of patient and caregiver's CBI.

**Patients**		**Caregiver**	
**Variables**	**Median values**	**Variables**	**Median values**

MMSE total score (0–30)	18.6 ± sd	CBI total score (0–96)	45.5 ± sd

ADL total score (0–6)	4.2 ± sd		

IADL total	2.7 ± sd male		
score (0–8 male 0–5 female)	2.8 ± sd female		

## Discussion

Data emerging from the present study indicate that the quality of life of the informal caregivers is closely correlated with the severity of the behavioural disorders and to the duration of the disease. Most of the subjects interviewed had to change many of their own habits to meet the needs of the in-patient living in the family. This situation of forced adaptation causes problems not only of an economic nature, due to the expensive care needed by the patient, but also in family life since the entire nucleus has to adapt to the single patient, in these situations [7-12]. Hostility towards relatives represents another variable that negatively affects the health of the person who *in primis *takes care of the patient, and this is also a source of stress. The most serious problem concerning the large number of caregivers that consistently suffer from anxiety and depression is that this situation stems from the overwhelming responsibilities related to the role that they cover [12,13]. The burden of responsibility significantly increases in relationship to the complexity of the disease and our study, in accordance with the national and international scientific literature, clearly confirms that the depressive state of the caregiver depends on the severity of the **AD **[6,14-16], thus demonstrating that the psychological condition, and, therefore, anxiety and depression of the caregiver, are closely related to the values of the MMSE, ADL, IADL and to the several phases of the cognitive and physical deterioration process of the patient [16,17]; as in the case of the depression, the caregiver's condition is also linked to social situations, such as income [16]. However, it has been emphasized that some of the tension between the patient and caregiver stems from the type of relationship that existed before the onset of AD [18].

## Conclusion

It has been well established that AD is a disease that involves not only the patient, but also affects the whole family. The complexity of the treatment, the constant commitment of the person taking care of the AD patient, as well as the inadequacy of the public service; the consequent effects upon the emotional and interpersonal relations; the direct and indirect costs of care also play a major role in influencing the social, psychological and physical wellbeing of the caregiver and of his/her family. Caring is held to be very demanding and emotionally involving. The main complaint of caregivers is the lack of support from the Public Health Service [7-18]. The continuous commitment in caring also leads to health problems and depression that has negative repercussions on the family. AD should not be considered exclusively as a health problem, but, most important, it is a social problem. The present research highlights and confirms fundamental data: the severity of the disease plays the most important role in the re-organization of the family structure, in those families that have to take care of patients who are not hospitalized. It is now mandatory to improve public facilities able to offer valid emotional and financial support, and help informal caregivers to tolerate the burden of caring, increasing the quality of the service [19]. It is vital to promote interventions able to reduce the strong impact that the AD patient has on his/her own caregiver; these initiatives should improve the quality of life of the patient. When planning these interventions, the physical, psychological and economic aspects of the patient and his/her caregiver must be taken into consideration. The planning and putting into practice of support interventions, guidance and aid to families could be a valid solution to the loneliness and the consequent care burden experienced by the family and the caregiver [17-19].

## Abbreviations

AD: Alzheimer's Disease; CNS: Central Nervous System; MMSE: Mini Mental State Examination; ADL: Activities of Daily Living; IADL: Instrumental Activities of Daily Living; CBI: Caregiver Burden Inventory

## Competing interests

The authors declare that they have no competing interests.

## Authors' contributions

MF, EDV, LDC, CB contributed to the design and coordination of the study. All contributed to the analysis of the questionnaires. TDB was responsible for collection of data. MF and TDB performed the statistical analysis. EL helped in the design of the questionnaires and drafting of the manuscript. All authors read and approved the final manuscript.
